# Towards an understanding of the enzymatic degradation of complex plant mannan structures

**DOI:** 10.1007/s11274-023-03753-7

**Published:** 2023-09-09

**Authors:** Mpho Stephen Mafa, Samkelo Malgas

**Affiliations:** 1 Carbohydrates and Enzymology Laboratory (CHEM-LAB), Department of Plant Sciences, University of the Free State, Bloemfontein, 9300 South Africa; 2 Department of Biochemistry, Genetics and Microbiology, University of Pretoria, Hatfield, 0028 South Africa

**Keywords:** Carbohydrate esterase, Glycoside hydrolase, Lytic polysaccharide monooxygenase, Mannan, Synergy

## Abstract

Plant cell walls are composed of a heterogeneous mixture of polysaccharides that require several different enzymes to degrade. These enzymes are important for a variety of biotechnological processes, from biofuel production to food processing. Several classical mannanolytic enzyme functions of glycoside hydrolases (GH), such as β-mannanase, β-mannosidase and α-galactosidase activities, are helpful for efficient mannan hydrolysis. In this light, we bring three enzymes into the model of mannan degradation that have received little or no attention. By linking their three-dimensional structures and substrate specificities, we have predicted the interactions and cooperativity of these novel enzymes with classical mannanolytic enzymes for efficient mannan hydrolysis. The novel exo-β-1,4-mannobiohydrolases are indispensable for the production of mannobiose from the terminal ends of mannans, this product being the preferred product for short-chain mannooligosaccharides (MOS)-specific β-mannosidases. Second, the side-chain cleaving enzymes, acetyl mannan esterases (AcME), remove acetyl decorations on mannan that would have hindered backbone cleaving enzymes, while the backbone cleaving enzymes liberate MOS, which are preferred substrates of the debranching and sidechain cleaving enzymes. The nonhydrolytic expansins and swollenins disrupt the crystalline regions of the biomass, improving their accessibility for AcME and GH activities. Finally, lytic polysaccharide monooxygenases have also been implicated in promoting the degradation of lignocellulosic biomass or mannan degradation by classical mannanolytic enzymes, possibly by disrupting adsorbed mannan residues. Modelling effective enzymatic mannan degradation has implications for improving the saccharification of biomass for the synthesis of value-added and upcycling of lignocellulosic wastes.

## Introduction

Advances in the formulation of enzyme cocktails that are effective in the saccharification step of lignocellulosic biomass, particularly the inclusion of xylanases and lytic polysaccharide monooxygenases (LPMOs) in cellulase cocktails, are helping cellulosic-ethanol biorefineries move towards commercial feasibility (van Dyk and Pletschke 2012; Malgas et al. 2019). However, these advances have mainly benefited the effective saccharification of lignocellulosic feedstocks containing xylans, such as agricultural residues (Beukes et al. 2008; Beukes and Pletschke 2011; Olver et al. 2011) and hardwoods (Malgas et al. 2017). Such advances have not been as significant in cocktail formulations for the saccharification of mannan-containing feedstocks, such as softwoods and spent coffee grounds.

Over the past several years, significant strides have been made in understanding the enzymology of the degradation and saccharification of plant mannans (Malgas et al. 2015a). During the same time, the discovery and description of new types of mannanolytic enzymes, such as mannobiohydrolases (Tsukagoshi et al. 2014a) and glucomannanases (Busch et al. 2019), have been made in numerous microorganisms, and understanding of the mechanistic behaviour of these enzymes is also gaining ground. The implication of nonhydrolytic proteins such as lytic polysaccharide monooxygenases (LPMOs) in the deconstruction of mannan has also been made recently (Fanuel et al. 2017). However, it is still unclear how microorganisms utilise these various proteins to effectively deconstruct mannans to serve them as carbon sources.

In this review, we summarize recent studies on enzymatic mannan degradation and infer how the classification of “classical” mannanolytic enzymes, such as β-mannanase, β-mannosidase and α-galactosidase, and auxiliary activity (AA) enzymes (LPMOs, swollenins or expansins and carbohydrate esterases (CE)) according to the CAZy database (http://www.cazy.org/) and their synergistic interactions during mannan degradation can be exploited for industrial applications involving mannan-containing lignocellulosic feedstocks. Elucidating up-to-date possible strategies for enzymatic degradation of mannan to oligosaccharides and monosaccharides can lead to improved production of value-added products, such as ethanol, prebiotic oligosaccharides, and artificial sweeteners, to which these enzyme breakdown products serve as precursors.

### The plant mannan structure and its role

**Fig. 1:**
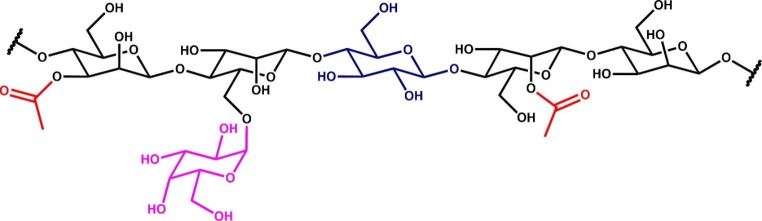
General structure of heteromannan, *O*-acetyl-galactoglucomannan. *O*-Acetyl-galactoglucomannans contain β-1,4-linked D-mannose residues (black) and also β-1,4-linked D-glucose residues (purple). This backbone is decorated with acetyl groups (red) at the 2- and 3-positions with α-linked D-galactosyl residues (cyan) at the 6-position of excusively mannose residues

Mannan, a type of hemicellulose, is separated into four groups, depending on which sugar(s) the β-1,4-linked backbone contains and the amount of α-1,6-linked galactose residues present (Sachslehner et al. 2000). These four groups of mannans are linear mannan, glucomannan (GM), galactomannan (GalM) and galactoglucomannan (GGM) (van Zyl et al. 2010; Malgas et al. 2015a). The β-1,4-linked backbones of linear mannan and GalM exclusively contain D-mannose, while that of GM and GGM contain both D-mannose and D-glucose (van Zyl et al. 2010). GalM generally contains more than 5% (w/w) D-galactose, while GGM is GM that contains more than 5% (w/w) D-galactose (van Zyl et al. 2010). GM and GGM are esterified with *O*-acetyl groups at the C2 and C3 positions of the hexoses that make up the mannan backbone (Fig. 1)(Bååth et al. 2018; Berglund et al. 2019).

Two main roles have been assigned to mannans: (i) structural, as paracrystalline fibrils, that support or most likely a substitute for cellulose as the primary structural polysaccharide of the cell wall, or cross-linking polymers that bind cellulose (Moreira and Filho 2008); and (ii) as storage reserves in the walls and vacuoles of seed endosperm, as well as the walls of vegetative tissue (Yamabhai et al. 2016). The high resistance of plant biomass to microbial degradation is often attributed to the presence of extractives and lignin, which covalently cross-links other polymers, such as hemicellulose (Várnai et al. 2011). The tight interactions of lignin with wood polysaccharides make the structure of the lignified cell wall so compact that molecules in the size range of proteins cannot easily penetrate them.

Numerous studies have investigated the supramolecular architecture and organization of the polymeric components in softwood secondary cell walls, which are GGM-rich. A rigid GGM population directly interacts with the cellulose surfaces (Berglund et al. 2020), mediated by the higher content of Glc in the backbone in this population, and the presence of even motifs of alternating Man units with higher content of Gal substitutions (Martínez-Abad et al. 2020). Molecular dynamics simulation studies suggested that GGM can bind stably to some hydrophilic faces and hydrophobic faces of cellulose microfibrils in plant cell walls via the Glc-to-Man-rich motif compared to the Man-rich motif of the GGM polysaccharide (Yu et al. 2018; Martínez-Abad et al. 2020).

On the other hand, a matrix mannan population, rich in acetylation, does not directly bond to cellulose but interacts covalently with lignin to form lignin-carbohydrate complexes (LCCs) (Martínez-Abad et al. 2020; Kirui et al. 2022). The links in softwood LCCs involve mainly mannan-lignin interactions through benzyl, ester, and phenyl glycosidic bonds and hemiacetal/acetal links (Tarasov et al. 2018). Lignin and LCCs are expected to limit the elastic deformation of lignified cell walls (Berglund et al. 2020). Benzyl ester bonds connect lignin and carbohydrate moieties through uronic acid side chains in xylan, while acetal bonds are through carbonyl groups of structural fragments of phenylpropane of lignin and hydroxyl groups of carbohydrates (Tarasov et al. 2018).

**Fig. 2:**
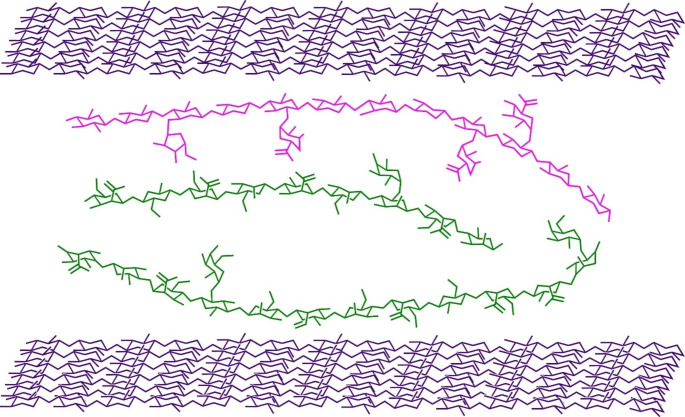
A conceptual scheme on how heteromannans interact (green) with other lignocellulosic fractions, such as cellulose (purple) and *O*-acetyl*-*arabinoglucuronoxylan (cyan). The lsHM such as GM regions binds to the cellulose microfibril surfaces and the hsHM binds to lignin (not shown) but not cellulose, while *O*-acetyl*-*arabinoglucuronoxylan hydrogen bond to the hydrophilic surfaces of cellulose through folding as a twofold helical screw. The hemicelluloses, *O*-acetyl-galactoglucomannan and *O*-acetyl*-*arabinoglucuronoxylan, may adopt more coiled conformations where they can interact with each other through bridging adhesion of different intensities. Finally, the ferulic groups attached to the arabinosly residues of the xylan enable coupling of xylan with lignin (not shown)

Furthermore, it is reported that the association occurs between the unsubstituted (“smooth”) regions of the mannan backbone or the low-substituted heteromannan (lsHM), and it would be blocked by galactose side chains in the densely substituted (“hairy”) regions of these polymer chains (Dhawan and Kaur 2007). The hetero-mannans whose main chain is less substituted by galactose units interact more among themselves (hyperentanglement) or with other biopolymers forming a loose network (Fig. 2). In flexible “hairy” or high-substituted hetero-mannan regions (hsHM), hemicelluloses can adopt more coiled conformations where they can interact with each other by bridging adhesion of different intensities, creating aggregated layers that can bridge adjacent cellulose bundles (Berglund et al. 2020).

### Mannan degradation

Mannan degradation is primarily facilitated by glycoside hydrolases (glycosidases, GHs), which are responsible for the cleavage of *O*-glycosides between sugar moieties and AA enzymes (Malgas et al. 2015a); (1) non-enzymatic proteins (swollenins and expansins), involved in fibre swelling and fragmentation of polysaccharide aggregations into short fibres (Yennawar et al. 2006; Herburger et al. 2020), (2) LPMO and CE, which catalyse the oxidative cleavage of glycosidic bonds and removal of ester linkages (Biely 2012; Agger et al. 2014), respectively.

### Mannanolytic glycoside hydrolases

#### Endo-β-1,4-mannanases

Studies on GH5 β-mannanases have shown that these enzymes require a minimum of four binding subsites to ensure productive binding to the substrate (Srivastava and Kapoor 2017). This was shown by studies in PaMan5A derived from *Podospora anserina* and TrMan5A derived from *Trichoderma reesei*, respectively, which showed that the enzymes could not efficiently cleave mannotriose (M3), but could cleave mannotetraose (M4) and mannopentaose (M5) more efficiently (Harjunpää et al. 1999; Couturier et al. 2013). It has been shown that GH5 β-mannanases show a higher affinity for GMs due to a relaxed specificity for glucose and mannose (M1) at the − 2 and + 1 subsites, where cleavage occurs, which means that the enzymes can efficiently cleave either of the two sugars at these positions (Tailford et al. 2009; Srivastava and Kapoor 2017). GH5 β-mannanases are known to possess transglycosylation activity. Transglycosylation occurs when a carbohydrate hydroxyl group from the substrate acts as an electron acceptor instead of water, as is often the case during substrate hydrolysis. This results in an oligosaccharide that has a higher degree of polymerisation (DP) than the original substrate (Couturier et al. 2013). Therefore, transglycosylation leads to decreased amounts of reducing sugars (RS) in the reaction resulting from the polymerisation of substrate hydrolysis products (Klyosov et al. 2012).

GH26 mannanases generally have six substrate subsites; +2 to -4, another study reported the presence of the subsite − 5 in the crystal structure of a *Bacteroides ovatus-derived* GH26 mannanase, *Bo*Man26B (Bågenholm et al. 2019). The crystal structure of *Bo*Man26B has a long, open active site cleft containing Trp-112 in subsite − 5 which is crucial for the binding of mannosyl groups (Bågenholm et al. 2016, 2019). Kaira and co-workers showed that Bacillus sp. GH26 mannanases have conserved substrate subsites that allow them to interact with substrates that have six sugars but require four sugars for efficient hydrolysis (Kaira et al. 2019). Subsites − 1 and − 2 play an important role in glucomannan and galactomannan hydrolysis, while subsites + 1 and + 2 are important in the interaction of the enzyme with unsubstituted mannan (Kaira and Kapoor 2019). *Bo*Man26B is more efficient in hydrolyzing GG than LBG (Bågenholm et al. 2019). These findings are consistent with those from a recent study where *Yunnania penicillata*-derived *Ypen*Man26A was more effective on GG than on LBG (Freiesleben et al. 2019). However, these findings were contrary to those of Kaira and Kapoor, who found that a *Bacillus sp.*-derived mannanase had high affinity and acted more efficiently on less substituted carob galactomannan than the more substituted GG (Kaira et al. 2019). Hydrolysis of mannan substrates by GH26 mannanases results in the production of mannooligosaccharides (MOS). Hydrolysis of mannohexaose (M6) by a GH26 mannanase of *Podospora anserine*, PaMan26A, resulted in the production of mannobiose (M2) and M4 as predominant sugars; while hydrolysis of M5 resulted in the production of M1 and M4 (Couturier et al. 2013).

The phytophagous beetles, mainly species from the super-families Chrysomeloidea and Laptinotarsa, harbour bifunctional GH family 45 gluco-mannanases which can degrade GM and amorphous cellulose (Kirsch et al. 2012; Busch et al. 2019). Interestingly, these enzymes show no activity in crystalline cellulose and GalM, but they release oligosaccharides with a DP of 2 to 4 during hydrolysis of carboxymethylcellulose and konjac GM (Busch et al. 2019). Unfortunately, none of these beetle-derived gluco-mannanases has solved three-dimensional structures. Therefore, no information about their mechanistic action is available.

GH family 113 only has three β-mannanases which have their structures to date; *Aa*ManA (3CIV) from *Alicyclobacillus acidocaldarius*, *Ba*Man113 (7DV7) from *Bacillus* sp. N-16-5 and *Ax*Man113A (5YLH) of *Amphibacillus xylanus* (Zhang et al. 2008; You et al. 2018b). When hydrolysing mannans, GH113 β-mannanases show the highest activity on the unsubstituted konjac GM than that against GalM, with LBG being preferred compared to GG and linear mannan (Zhang et al. 2008; You et al. 2018b), except for *Ba*Man113, which shows similar activity between GM and LBG (Liu et al. 2021). The enzymatic activity of the β-mannanases is limited by the galactose side groups in GalM and poorly hydrolyses the glycosidic linkages in crystalline and insoluble substrates such as linear mannans (You et al. 2018b). This could possibly due to the lack of crystalline biomass-specific CBMs which can disrupt the structural integrity of the polysaccharide for catalysis to take place. Interestingly, the smallest MOS that *Ax*Man113A and *Ba*Man113 hydrolyse is M2, while M3 is the smallest MOS *Aa*ManA is active on, however, all these enzymes generally display increased velocity when hydrolysing MOS with DP higher than 3 (Zhang et al. 2008; You et al. 2018b).

GH134 β-mannanases are the only family that mechanistically operates via a single-displacement reaction with inversion of the anomeric configuration (www.cazy.org/GH134.html). In this case, reactions require the participation of a general acid and a general base with a nucleophilic attack by a molecule of water (Jin et al. 2016). To date, only three mannanases have been biochemically characterized in this family; the *Streptomyces sp.* NRRL B-24,484 derived *Ss*GH134, *Rhizopus microsporus* (*Rm*Man134A) and *Aspergillus nidulans* derived *An*Man134A (www.cazy.org/GH134.html). *An*Man134A released M2, M3, and M4, with M3 being the predominant reaction product, when acting on α-galactosidase de-branched GalM (Shimizu et al. 2015). Because no M1, M5 and M6 were produced, this suggests that *An*Man134A employs an initial endolytic attack followed by processive hydrolysis which releases M3 (Shimizu et al. 2015). Similarly, *Ss*GH134 hydrolysed MOS with a DP greater than 5, yielding predominantly M3, with smaller amounts of M2 and M4 (Jin et al. 2016), while *Rm*Man134A could not hydrolyse MOS with DP ≤ 4 (You et al. 2018a).

#### Exo-β-1,4-mannobiohydrolases

Over the past decade, a new mannanolytic enzyme class has been discovered and is suggested to be implicated in the efficient degradation of the mannan backbone, this enzyme class is called exo-β-1,4-mannobiohydrolase (EC 3.2.1.100). According to the CAZy database, only three exo-β-1,4-mannobiohydrolases (*Bacteroides ovatus Bo*Man26A, *Cellvibrio japonicas Cj*Man26C, and *Reticulitermes speratus Rs*Man26H) have been characterised to date (www.cazy.org). Mannobiohydrolases are responsible for the hydrolysis of β-1,4-D-mannosidic linkages in β-D-mannans, resulting in the removal of successive M2 residues from the non-reducing chain ends of mannans (Cartmell et al. 2008; Tsukagoshi et al. 2014b; Bågenholm et al. 2016).

#### β-mannosidases

β-Mannosidases (EC 3.2.1.25) catalyse the release of mannose units from MOS or in some cases mannans, from the terminal non-reducing ends of the substrates (Yeoman et al. 2010; Xie et al. 2019; Kalyani et al. 2021). Mannosidases are classified under the GH families, 1, 2, 5, 113 and 164 based on their sequence and structural similarities. Enzymes belonging to other families are well characterised, except those that belong to GH families 113 and 164, whose characteristics are still not understood. It was revealed that a Firmicutes-derived GH113 mannosidase did not have transglycosylation activity like those classified in families 1, 2, and 5; the second distinguishing characteristic was that the enzyme was active on numerous mannans, such as Konjac GM, carob, and Guar GalM (Couturier et al. 2022). In the case of GH164 mannosidases, it was revealed that a *Bacteroides salyersiae*-derived β-mannosidase only hydrolysed short MOS (Armstrong and Davies 2020). The authors did not test the activity of this enzyme on polymeric mannan substrates; however, they showed that it exists as a doughnut-shaped homotrimer in solution, which is a unique structural conformation for mannosidases (Armstrong and Davies 2020).

#### α-galactosidases

α-Galactosidases, also called melibiases (EC 3.2.1.22), are exo-acting enzymes that cleave terminal nonreducing galactose residues from α-D-galactose-containing oligosaccharides, such as melibiose, raffinose, and stachyose, and polysaccharides. α-Galactosidases are classified into GH families 4, 27, 31, 36, 57, 97 and 110, according to the CAZy database. Generally, the GH27 galactosidases act on galactomannan polymers and galactose-containing oligomers, while GH 36 α-galactosidases are specific towards galactose-containing oligomers (Malgas et al. 2015b). Interestingly, BT3661, a GH97 galactosidase from *Bacteroides thetaitaomicrom*, catalyses the hydrolysis of both α-galactoside and β-L-arabinofuranoside residues from substrates (Kikuchi et al. 2017). The GH110 counterparts are active on α-1,3-linked galactose residues in polysaccharides such as λ-carrageenan (Anisha 2022).

#### Endoglucanases

Endoglucanases (EC 3.2.1.4) catalyse the endo-hydrolysis of β-D-1,4-linkages at amorphous sites of cellulose chains. Interestingly, several studies have shown that some endoglucanases can cleave the β-D-1,4-glycosidic bond between glucopyranosyl and mannopyranosyl units in GM. Another study demonstrated that endoglucases, Cel5A and Cel7B, soured from *T. reesei*, hydrolysed Konjac GM to produce DP 2–4 mannooligosaccharides and gluco-mannooligosaccharides; GM1 and GM2 (Mikkelson et al. 2013). Miao et al. also showed that *Aureobasidium pullulans*-derived endoglucanase (ApCel5A) catalysed the production of glucose, M2 and M3 from Konjac GM hydrolysis (Miao et al. 2021).

#### β-glucosidases

β-Glucosidases (EC 3.2.1.21) catalyse the hydrolysis of terminal, non-reducing β-D-glucosyl residues with the release of β-D-glucose from cellulose and GM (Jäger et al. 2010; Bai et al. 2021). β-Glucosidases are classified into GH families 1, 3, 5, 9, and 30; with GH1, 3, 5 and 30 β-glucosidases falling into GH Clan A, which consists of proteins with (β/α)_8_-barrel structures, while GH9 glucosidases have (α/α)_6_-barrel structures (www.cazy.org).

### Auxiliary activity enzymes

#### Acetylmannan esterases

Acetylmannan esterases (AcMEs; EC 3.1.1.6) are responsible for the deacetylation of 2- or 3-O-acetylated mannopyranosyl residues and the release of acetyl groups. AcMEs are classified under the CE superfamily, which consists of about 20 families and one unclassified family containing 2756 GenBank accession numbers (CAZy database: 27/05/2023). Few of the 20 CE families have mannan deacetylation activities that remove acetic acids, such as CE families 1, 2, 4, 5, 6, 12, and 16. A recent study argue that CE1 and CE5 are well studied, but CE2, CE4, CE6, CE7 and CE16 were not thoroughly studied (Venegas et al. 2022). In addition, a few studies have investigated the CE action towards specific acetylated positions within mannan substrates (Mai-Gisondi et al. 2017).

Using polygenetic analysis, CE16 has been divided into four groups based on amino acid sequence similarity (Venegas et al. 2022). The authors studied four enzymes sourced from *Aspergillus niger* NRRL3 called Hae-A, Hae-B, Hae-C, and Hae-D, which showed different substrate specificities. The Hae-A enzyme displayed deacetylation activity, which released 70 to 80% acetic acid from acetylated mannan and MOS. Hae-C and Hae-D had residual deacetylation activities on acetylated mannan and MOS, releasing less than 20% acetic acid from both substrates. The finding reveals that Hae-A was the only enzyme with efficient mannan deacetylation activity.

Three acetylxylan esterase enzymes from *Aspergillus nidulans*; *An*AcXE (CE1), *Orpinomyces* sp., *Os*AcXE (CE6), and *Myceliophthora thermophila*, *Mt*AcE (CE16), had varying esterase activity towards acetyl-GGM (Mai-Gisondi et al. 2017). Regional specificity studies revealed that the positional preferences of *Os*AcXE and *Mt*AcE were more similar when studied with 2-O-acetyl-Man*p* substituents, while the activity of *An*AcXE was significantly higher towards 3-O-acetyl-Manp substituents (Mai-Gisondi et al. 2017). In addition, CE2 and CE17 were demonstrated to be highly specific toward mannan substrates (Michalak et al. 2020). Two acMEs sourced from the human gut bacteria *Roseburia intestinalis* showed varied acME activity, with RiCE2 removing 3-O-, 4-O-, and 6-O-acetylations, while *Ri*CE17 only demonstrated the region-specificity of 2-O-acetylation (Michalak et al. 2020). The synergistic activities of *Ri*CE17 and *Ri*CE2 completely removed the acetyl groups from several mannans and MOS.

Some acMEs have not yet been classified into carbohydrate esterase families, but their physicochemical properties are well established (Pawar et al. 2013; Saito et al. 2022). Two esterases from *Aspergillus oryzae* RIB40 (rAME1 and rAME2) showed different activities on mannan polymers and MOS. rAME2 hydrolysed KGM and MOS, but rAME1 only showed activity on MOS substrates. Acetyl release by rAME2 was 100% and 80% from MOS and KGM, respectively, while rAME1 released 60% acetyl from MOS. rAME1 had the propensity to act on the single acetyl substitutions at 2-O and 3-O positions, while double substitutions were not removed (Saito et al. 2022). It has been shown that some CE1 to CE7 and CH16 enzymes had broad hemicellulose activity (previously assigned as acetyl-xylan esterases) (Pawar et al. 2013). However, there is no significant information on acetyl-GM in the literature. But the acetylation positions on the acetyl-GM and acetyl-glucuronoxylan are similar (Biely 2012). However, the OH-2 (hydroxyl group) on mannopyranosyl residues is in the axial position compared to the equatorial position of xylopyranosyl residues (Biely 2012). The differences in the OH-2 orientation could explain the steric hindrance toward the CE2 and CEX (*Ri*CEX), which only improved their activity when they act in synergy or CE2 required CE17 synergistic action to improve de-acetylation of MOS or mannan. Lately, the similarities in the orientations of the acetyl groups attached to mannan and xylan substrates imply that some of the CE1 to CE7 can deacetylate mannan substrates.

#### Lytic polysaccharide monooxygenases

LPMOs are copper-containing AA enzymes that cleave polysaccharides in an oxidative manner (Forsberg et al. 2014). There are two types of cellulose-active LPMOs; C1-hydroxylating LPMOs (EC 1.14.99.54), which produce cellulose fragments that contain a residue of D-glucono-1,5-lactone at the reducing end, which hydrolyses quickly and spontaneously to aldonic acid, and C4 dehydrogenating LPMO (EC 1.14.99.56), which produce cellulose fragments that contain a residue of 4-dehydro-D-glucose at the nonreducing end (Mafa et al. 2021). C1-hydroxylating LPMOs are found in AA9,10 and 14, while C4-dehydrogenating LPMOs are found in AA9 and 10. Recently, enzymes with activity against non-crystalline (soluble) polysaccharides and oligomeric structures have been identified among LPMOs (Liu et al. 2018; Petrović et al. 2019).

Petrovic et al. (2019) recently characterized three cellulose-active C4-oxidizing family AA9 LPMOs from the fungus *Neurospora crassa*, *Nc*LPMO9A (NCU02240), *Nc*LPMO9C (NCU02916), and *Nc*LPMO9D (NCU01050). They showed that all three LPMOs were active on konjac GM, furthermore, showed that the activity on KGM was promoted when KGM was coated on phosphoric acid swollen acid cellulose (PASC), in particular for *Nc*LPMO9D (Petrović et al. 2019). Interestingly, no activity for any LPMO was observed toward ivory nut mannan, either in the absence or in the presence of PASC (Petrović et al. 2019). A previous study also showed that *Nc*LPMO9C requires short stretches of contiguous β-1,4-linked glucose units for activity, hence the lack of activity in carob GalM (Agger et al. 2014). Another study revealed that *Hi*LPMO9I from the white-rot conifer pathogen *Heterobasidion irregulare* displayed cleavage activity against GM (Liu et al. 2018). Similar to the C4-oxidizing activity of *N. crassa*-derived LPMOs, *Hi*LPMO9I produced C4-oxidized sugar products with a DP of 3–5.

On the other hand, the *Podospora anserina*-derived *Pa*LPMO9H catalyses C1/C4-oxidative cleavage of GM (Fanuel et al. 2017). Recently, an LPMO from *Pleurotus ostreatus* (*Po*LPMO9D) was shown to efficiently depolymerise GM and produce a wide range of oligomers with a DP of 3–12, which were a mixture of neutral and C1/C4-oxidized glucomannan-oligomers (Li et al. 2021). A recent study showed that a novel AA10 LPMO derived from *Bacillus subtilis* (*Bs*LPMO10A) exhibits an extensive active-substrate spectrum, particularly for polysaccharides linked via β-1,4 glycosidic bonds, such as β-(Man1 → 4Man); LBG and KGM (Sun et al. 2023).

#### Expansins and swollenins

Hemicelluloses can bond cellulose microfibrils together, forming a strong load-bearing network. Expansin (EXP) is thought to disrupt the cellulose-hemicellulose association transiently, allowing slippage or movement of cell wall polymers before the association reforms and the integrity of the cell wall network is re-established (Mafa et al. 2021). EXPs are also implicated in other plant developmental processes where cell wall loosening occurs, such as in fruit softening, organ abscission, seed germination, and pollen tube invasion of the grass stigma (Yennawar et al. 2006). Two expansin families with wall-loosening activity have been identified in land plants, named α-expansins (EXPA) and β-expansins (EXPB) (Herburger et al. 2020). Expansins share a bidomain structure, with domain 1 homologous to fungal GH45 β-1,4-endoglucanases, while domain 2 of these proteins are homologues to group-2 grass pollen allergens (Herburger et al. 2020). Due to the presence of several aromatic residues on the protein surface, expansin domain 2 has been proposed to resemble the cellulose-binding domain of cellulases (Andberg et al. 2015). Due to its unique action, numerous studies have implicated expansin in the enhancement of CAZyme activity during the hydrolysis of cellulose/lignocellulosic biomass.

Fungal organisms also possess another non-hydrolytic protein called swollenin, which is similar to the expansins in its action. Swollenins are reported to modify the chemistry and structure of microcrystalline polysaccharides in lignocellulose by reducing its degree of crystallinity, creating more binding and cleavage sites, thus allowing CAZymes to hydrolyse polysaccharides effectively. As a result of their specificity, swollenins can disrupt polysaccharide structures at the microscopic level without detectable RS release and lead to bulk microcrystalline polysaccharide swelling. Fungal swollenins have sequence similarity to expansins and are often referred to as expansin-like proteins.

It has been shown that a bacterial expansin (BsEXLX1) binds to lignin strongly, whereas it showed similar binding to Avicel and xylan substrates (Xu et al. 2023). It has also been shown that a *Trichoderma pseudokoningii* S38 swollenin (SWO I-P) and *T. reesei* SWO I-R both had subtle activity on xylan and yeast cell wall glucan (Yao et al. 2008). Finally, a recent study showed a swollenin released xylose and xylotriose when acting alone, while it showed little synergism when combined with the cellulase mono-components exoglucanase (Cel7A) and endoglucanase (Cel5A), but showed pronounced synergism with xylanase mono-components from GH10 and GH11, resulting in the release of significantly more xylose (> 300%) from steam-pretreated corn stover (Gourlay et al. 2013). These non-hydrolytic proteins induce the disruption or amorphogenesis in the bulk crystalline, insoluble holocellulose fraction, which is the total polysaccharide fraction of biomass. According to these three studies, expansins and swollenins may also interact with hemicellulosic substrates such as mannans.

#### Carbohydrate binding modules

Carbohydrate binding modules (CBMs) are noncatalytic domains appended to catalytic proteins or scaffoldin subunits in multienzyme extracellular complexes, such as cellulosomes. The role of CBMs is to localise the soluble enzyme to its target substrate, and in some cases, it is also suggested that CBMs can alter the structural integrity of the polysaccharide matrix in biomass, making it more accessible to enzyme hydrolysis (Shallom and Shoham 2003; Shoseyov et al. 2006). There are three types of CBMs; namely Type A, Type B, and Type C modules. Type A CBMs are those that bind to the surfaces of crystalline polysaccharides and show little or no affinity for soluble carbohydrates (Boraston et al. 2004). Type B CBMs, on the other hand, interact with single polysaccharide chains and bind to polysaccharides that are the substrates for the cognate catalytic module of the enzyme (Boraston et al. 2004; Shoseyov et al. 2006). Lastly, Type C CBMs bind optimally to oligosaccharides (Boraston et al. 2004). The CBMs are classified into families, based on amino acid sequence similarity in the CAZy database.

### Synergistic action of GHs and AA enzymes during mannan degradation

#### Synergism between mannanolytic GHs

The synergistic actions which occur between mannanolytic GHs have been comprehensively reviewed recently by our lab (Malgas et al. 2015a). Synergistic associations between these enzymes are classified into two types; (1) homeosynergism, which is synergy between mannanase and mannosidase during the mannan backbone cleavage, and (2) heterosynergism, which is synergy between a backbone cleaving enzyme, such as mannanase or mannosidase, and a sidechain cleaving enzyme such as α-galactosidase (Malgas et al. 2015a). To date, numerous studies have evaluated the cooperative action between β-mannanases and α-galactosidases during GalM hydrolysis, with synergism detected in most of these studies, while a lack of synergy and/or antisynergy was observed in some cases. A recent study has shown that the cooperative effect between β-mannanase and α-galactosidase could shift from synergy to anti-synergy when increasing the ratio of α-galactosidase/β-mannanase (Hsu and Arioka 2020).

Interestingly, all synergy studies conducted on mannanolytic GHs have exclusively used only GH5 and 26 β-mannanases, while only GH2 and GH5 β-mannosidases, and GH27 and 36 α-galactosidases. A recent study showed synergism between β-mannanase, GH5_7 (sub-family 7), and β-mannosidase, GH2-1, from *Neurospora crassa* during hydrolysis of β-mannan (Hsu and Arioka 2020). The literature has generally shown that the GH5-derived mannosidases synergise with mannanases, while the GH2 mannosidases have been shown to either not synergize (Shi et al. 2011; Malgas et al. 2022) or anti-synergize with mannanases (Hägglund 2002; Shi et al. 2011). It should be noted that in vivo these two enzymes are not supposed to be localised in the same compartment, since GH5_7 is extracellular, while GH2_1 is intracellular (Hsu and Arioka 2020).

The synergism between mannanase and galactosidase in heteromannans is mainly attributed to the removal of galactose side chains by polymer-active GH27 galactosidases; this likely increases mannanase-polymer interactions (Malgas et al. 2015a). However, some exceptions have been reported in this regard; for example, a recent study showed that a GH36 galactosidase, AglB, was more active and synergised strongly with a mannanase on GalM (GG, carob, and LBG) hydrolysis, than GH27 counterparts; AglA, AglE, and AglF (Coconi Linares et al. 2020). Concerning synergistic galactose removal, no clear trends were observable among the combinations of mannanase to galactosidase applied, but it appeared that synergy was a result of the mannanase releasing oligomeric fragments from the GalM polymers that are preferred substrates for the oligomer-specific galactosidases such as those from GH36 (Coconi Linares et al. 2020).

Another recent study, with surprising results, showed that *Lichtheima ramosa* Man5B and Agal36B synergised the most during simultaneous application (+ 19% RS), followed by sequential application (first, AgalB, then Man5B) (+ 11% RS), while the inverse sequential application was antisynergistic (-8% RS) during palm kernel meal (Xie et al. 2019). These findings were unexpected since GH36 galactosidases are generally regarded as incapable of debranching galactose residues attached to polymers.

#### Synergism between mannanases and AcMEs

Effective hydrolysis of acetylated mannans requires the synergistic action of AcMEs and mannanases. The acetylation of mannans changes their solubility properties, making them insoluble (Bi et al. 2016; Bååth et al. 2018). As a result, a higher level of acetylation usually results in reduced activity of mannanases. Interestingly, supplementation of an esterase (CE2) from *Clostridium thermocellum* (*Ct*Axe2A) significantly increased the activity of *Cj*Man5A by approximately 30% during KGM saccharification (Bååth et al. 2018). On the other hand, the synergy between *Cj*Man26A and *Ct*Axe2A only increased the saccharification yield of KGM by about 10%. From this study, it appeared that the GH26 mannanase, CjMan26A, was more tolerant to the acetylation in KGM compared to the GH5 CjMan5A enzyme. Another study used a mannanase from *Bacteroides ovatus* (*Bo*Man26B) to hydrolyse LBG and softwood mannan. The results showed that after *Bo*Man26B hydrolysis of softwood mannan, some generated DP 2–5 MOS were acetylated (Bhattacharya et al. 2021). It was also shown that an acetyl-GGM esterase from *Aspergillus oryzae* improved mannanase activity during Norway spruce degradation, resulting in more than 85% hydrolysis yield (Tenkanen et al. 1995). The findings in the aforementioned studies show that polysaccharide deacetylation is essential to achieve complete saccharification of mannan substrates; which supports the thesis that removal of acetyl decorations by acetyl-mannan esterase enzymes can help achieve higher saccharification yield levels by CAZymes.

#### Synergism between GHs and AA enzymes

To date, only one study has reported on the synergistic action of GHs and LPMOs during the degradation of mannans. A recent study showed that degradation of LBG after co-incubation of *Bs*LPMO10A and mannanase, *Bs*MAN26, for 72 h leads to a reduction of sugar increase of 11.68% when compared to hydrolysis of *BsMAN26* alone (Sun et al. 2023). To date, it seems that only LPMOs allocated in AA family 9 and 10 display catalytic activity toward mannans such as GM. It is also interesting to note that *Bs*LPMO10A is the only AA reported to exhibit catalytic activity on GalM, as most reported AA proteins are known to act on GM-type mannans. It would be interesting to conduct biodiscovery studies to see if more AA proteins display similar activity to *Bs*LPMO10A. Although no synergy studies have been conducted with the GM-specific AA9 LPMOs, based on their catalytic specificity, it is clear that they have the potential for application in the efficient degradation of feedstocks containing GM or GGM, such as hardwoods and softwoods, respectively.

#### Synergism between GHs and noncatalytic proteins (expansin and swollenin)

A recent study has shown the role of swollenins in improving the degradation of mannans by mannanolytic GHs. *Aspergillus fumigatus* HBFH5-derived swollenin, *Af*Swol, showed a strong synergistic interaction with the mannanase, *Af*Man5A, during LBG GalM degradation, increasing the release of sugars by up to 1.31-fold (Gu et al. 2021). Synergism between the two proteins during LBG hydrolysis was obtained during both simultaneous (*Af*Man5A and *Af*Swo1 added at the same time) and sequential application; first, *Af*Man5A, then *Af*Swo1, or first, *Af*Swo1, then *Af*Man5A. Interestingly, not only was a *T. reseei* swollenin (SWOI) shown to have activity on substrates containing β-1,4-glycosidic bonds, i.e. carboxymethyl cellulose, hydroxyethyl cellulose and β-glucan, but was also able to hydrolyse soluble cello-oligosaccharides and the products formed were all consistent with SWOI cleaving a cellobiose unit off the substrate (Andberg et al. 2015). Due to LBG’s partially soluble nature, it is possible that *Af*Swo1 might have not utilised its amorphogenesis activity during LBG degradation, but used its hydrolytic activity to aid *Af*Man5A synergism. Similarly, another recent study has shown that a noncatalytic protein, Athe_0181, from *Caldicellulosiruptor bescii*, synergises with a multifunctional GH, CelD (composed of the two catalytic domains; CbMan5C and Cel5A), during the degradation of the mannan-containing palm kernel meal (PKM), with synergistic activity reaching 80.1% (Zhu et al. 2022). Reaction mixtures with inactive protein were used as controls during the experiments. Therefore, the synergistic effect of Athe_0181 could not have resulted from the protein blocking non-productive binding sites on PKM or stabilising CelD, but from the protein’s ability to modify the crystalline portions of the bulk PKM biomass, making CelD more accessible to it.

#### C_1_-C_x_ intramolecular synergism in mannanase

Intramolecular synergism is distinct from the aforementioned intermolecular synergism between discrete protein molecules; this is the synergism between domains within a modular protein, such as a catalytic domain, denoted C_x_, and a CBM, denoted C_1_ (Din et al. 1994), connected by a flexible linker peptide (Shoseyov et al. 2006). Von Freiesleben and co-workers evaluated the influence CBMs on the action of mannanases against the GalM substrates; GG and LBG. Their study showed that the activity of the *T. reesei*-derived *Tr*Man5A was the same on LBG and GG irrespective of the presence of the CBM1(von Freiesleben et al. 2016). They alluded to this observance being under CBM1 binding affinity, which is specific for cellulose but not mannan (von Freiesleben et al. 2016). On the other hand, *Pa*Man26A, which contains CBM35, had a significantly higher initial rate on LBG compared to the *Pa*Man26A core, which is CBM35 truncated, while no differences in GG hydrolysis rates were observed. An explanation could be that CBM35 interacts with LBG by binding to the β-mannan backbone or α-galactopyranosyl residues. During the hydrolysis of softwood GGM, a *T. reesei* mannanase (*Tr*Man5A) with a CBM1 and *Collariella virescens* mannanase (*Cv*Man26A) with two CBMs (CBM35 and CBM1) showed higher catalytic activity compared to mannanases that only had a catalytic domain (von Freiesleben et al. 2018). The authors demonstrated that CBM1 was responsible for the improvement in mannanase activity as most mannanases with CBM35 showed significantly lower catalytic activity. The possible reason for the synergism between CBM1 and mannanase is that CBM1 targets crystalline cellulose and locates mannanase close to the mannan covering or intertwined with microcrystalline cellulose (von Freiesleben et al. 2018; Uechi et al. 2020).

#### A proposed up‑to‑date model of mannan degradation

On review of the literature on the enzymatic degradation of mannans, we present an up-to-date model on how mannanolytic enzymes mechanistically degrade complex mannans (i.e., *O*-acetyl-GGM) in this review. First, GH5 and 113 mannanases and GH45 gluco-mannanases preferably cleave unsubstituted regions of the mannan backbone or glucomannans (von Freiesleben et al. 2016; Freiesleben et al. 2018; You et al. 2018b). The promiscuity of the gluco-mannanases may be indispensable for the hydrolysis of the cellulose-to-mannan junctions formed by lsHM motifs coating cellulose fibres. The mannanases may generally be sterically hindered by the presence of acetyl groups on the mannopyranosides constituting the mannan backbone; this then necessitates the action of acetyl mannan-specific esterases to remove these groups on the mannan backbone to allow mannanase action to proceed (Bååth et al. 2018).

Second, GH26 and 134 mannanases can proceed to cleave highly decorated GGM backbones (hsHM) or the soluble MOS generated from the insoluble lsHM motifs by the GM and lsHM-specific mannanases. hsHM polymers can cause steric hindrance of mannanase action, particularly block-wise substituted regions, such as those found in guar gum (Mccleary et al. 1985; Dea et al. 1986; Daas et al. 2000), thus necessitating the action of polymer-specific galactosidases, such as those of GH27, to remove excess galactose substitutions on hsHM (Malgas et al. 2015b). This may lead to an improved action of mannanase in these regions; however, excessive removal of galactose from lsHM may lead to hyperentanglement/aggregation of the polymers, leading to their precipitation or insolubility (Reddy et al. 2016). The mannanase-released mannooligosaccharides, from HsHM, which may be galactose substituted can be acted upon by the GH5 exo-mannanases and mannosidases, which can tolerate these substituents during the processing of mannooligosaccharides (Dias et al. 2004; Malgas et al. 2022). The galactose substituents remaining in these hsHM-generated MOS can also be acted upon by the GH36 galactosidases that have restricted substrate specificity to small galactose-containing oligosaccharides (Malgas et al. 2015a).

These aggregated lsHM polymers may be amenable to catalysis by AA9 LPMOs, which seem to show specificity toward insoluble GM-type mannan segments (Petrović et al. 2019). The linear mannan-rich segments of hyperentangled lsHM can be altered by the non-catalytic activity of expansin/swollenin, improving their solubility and accessibility by hydrolytic and lytic mannanolytic activities (Gu et al. 2021; Zhu et al. 2022). On the other hand, mannobiohydrolases would also be active on the amorphous/disrupted lsHM, processively releasing mannobiose residues from the nonreducing chain ends (Kawaguchi et al. 2014; Tsukagoshi et al. 2014a). The M2 residues would then be preferentially acted upon by the short DP mannooligosaccharide-active GH2 mannosidases (Tailford et al. 2007; Malgas et al. 2022). In the case of the generation of glucomannan-oligosaccharides, a glucosidase would be required to release glucose residues from the terminal, non-reducing β-D-glucosyl residues (Cairns and Esen 2010; Njokweni et al. 2012).

**Fig. 3:**
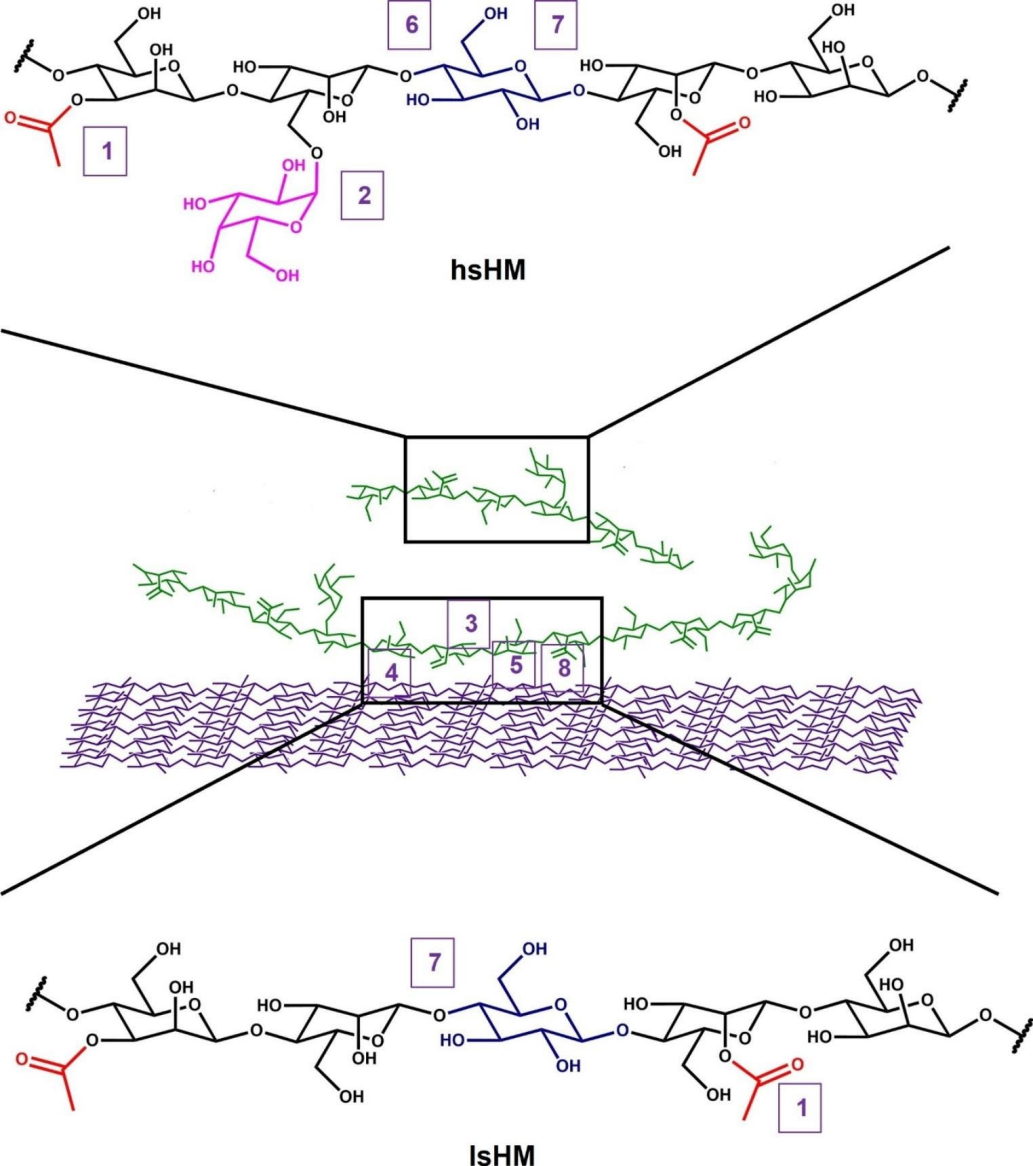
A general scheme of how hydrolytic mannanolytic enzymes mechanistically degrade hetero-mannans within lignocellulose in a synergistic fashion with the aid of non-GH proteins, such as CEs, CBMs, expansins, LPMOs and swollenins. The cellulose bound lsHM such as GM regions is degraded by the aid of (1) AcME that removes acetyl groups, (3) CBM may assist in directing key enzymes towards cellulose-mannan junctions, disruption of cellulose-mannan junctions is facilitated by (4) EXP, (5) LPMO and (8) SWO, and oxidative cleavage of mannan by (5) LPMO, and (7) MAN active on GM and linear mannan releases MOS and gluco-MOS. The water soluble hsHM region is degraded by the aid of (2) Aga that removes galactosyl substituents, (6) MBH removes successive mannobiose residues from the non-reducing ends of the mannan, and (7) MAN active on GalM and GM releases MOS, galacto-MOS and gluco-MOS from the mannan. Finally, AcME, Aga, BGL and Mnd act on solubilised *O*-acetylated MOS, galacto-MOS, gluco-MOS and MOS, respectively (not shown)

This review shows that the entire consortium of mannanolytic enzymes, including accessory/non-GH enzymes such as CEs, non-hydrolytic proteins (expansin and swollenin) and LPMOs, is required for the complete degradation of hetero-mannan. We have compiled a list of all the enzymes which, to date, are essential for the efficient degradation of *O*-acetyl GGM (see Table 1; Fig. 3). We believe that the aforementioned model of mannan degradation sheds insight into the selection of not only the necessary enzyme classes required but also the specific families described in the CAZy database and the rational application of these enzymes in enzyme cocktails to achieve high yields of VAP production from mannans and biomass containing mannan. This should lead to a significant improvement in the economic viability of the bioconversion of mannan-containing lignocellulosic biomass into various VAPs, as higher saccharification yields and lower protein dosages could be achieved.

**Table 1:** Key enzymes suggested for efficient degradation of *O*-acetyl-galactoglucomannan

Enzyme class (EC number)	CAZyme family	Substrate Specificity	References
AcME (EC 3.1.1.72)	CE2, 16	Active on 3-*O*-, 4-*O*- and 6-*O*-acetylations on hetero-mannans	(Bååth et al. 2018)
	CE17	Active on 2-*O*-acetylations, including double substituted oligomers	(Michalak et al. 2020)
Aga (EC 3.2.1.22)	GH27	Active on both short MOS and mannans substituted with D-galactose residues	(Malgas et al. 2015b; Coconi Linares et al. 2020)
	GH4, 36	Active on short MOS substituted with _D_-galactose residues	(Malgas et al. 2015b; Coconi Linares et al. 2020)
BGL (EC 3.2.1.21)	GH1, 3	Active in terminal, non-reducing _D_-glucosyl residues derived from glucomannan	(Bai et al. 2021)
CBM	CBM1	Affinity towards cellulose	(von Freiesleben et al. 2016; Freiesleben et al. 2018; Uechi et al. 2020)
	CBM35	Affinity towards mannans	(von Freiesleben et al. 2018)
EXP	-	Disruptor of cellulose-hemicellulose association	(Zhu et al. 2022)
LPMO (EC 1.14.99.54/56)	AA9, AA10	Disruption of GM-celluose complexes and oxidative cleavage of carbohydrates	(Sun et al. 2023)
MBH (EC 3.2.1.100)	GH26	Non-reducing end specific exo-mannanase removes successive mannobiose residues from mannan	(Cartmell et al. 2008; Reddy et al. 2016)
Mnd (EC 3.2.1.25)	GH1, 2, 164	Active on terminal, nonreducing D-mannose residues in short MOS (higher specificity with decreasing DP)	(Hsu and Arioka 2020; Armstrong and Davies 2020; Couturier et al. 2022)
	GH5	Active on terminal, non-reducing D-mannose residues in long MOS (higher specificity with increasing DP)	(Malgas et al. 2022)
MAN (EC 3.2.1.78)	GH5	Active on glucomannan and insoluble mannan	(Tailford et al. 2009)
	GH26	Active in GalM and soluble mannan	(Tailford et al. 2009)
	GH45	Active in GM and cellulose	(Kirsch et al. 2012; Busch et al. 2019)
	GH113, 134	Active on linear mannan	(You et al. 2018a, b)
Swol	-	Disruptor of cellulose-hemicellulose association	(Herburger et al. 2020; Gu et al. 2021; Zhu et al. 2022)

## Conclusions and future perspectives

The present review has shown that the complex structure of mannans poses a major challenge for enzymatic degradation. Analysis of the literature shows that mannan-specific GHs complemented by AA enzymes (CEs, expansins, swollenins, and LPMOs) are required for efficient mannan degradation. Therefore, the combined use of GHs and AA enzymes may increase the monosaccharide yield from mannan compared to using either enzyme alone during hydrolysis. Finally, a current model for mannan hydrolysis is proposed based on recent progress in deciphering the mechanism of action of each enzyme class.

Screening of new mannanolytic enzyme-producing microbes, mining of the enzyme coding sequences, genetic engineering of these enzymes and their large-scale production to complement enzyme cocktails are recommended for their commercial application in lignocellulosic biorefinery, especially for high-mannan feedstocks such as softwoods. Furthermore, studies should be conducted to understand the structure–function relationship and substrate recognition of the novel mannanolytic activities, particularly the AA10 LPMOs exhibiting GalM activity, gluco-mannanases and mannobiohydrolases that have not been evaluated in synergy studies with other mannanolytic enzymes during mannan degradation. In addition, a comprehensive characterisation of the CE families 1, 2, 4, 6, 7 and 17 may improve our understanding of their application in the removal of acetyl functional groups on mannan biomass. It is also apparent that endoglucanase, in synergy with mannanolytic enzymes catalysing GM, can produce novel gluco-mannan-oligosaccharides with prebiotic activity.

## Data Availability

Data sharing is not applicable to this article as no new data were created or analysed in this study.

## Abbreviations

AA: Auxiliary activity
Aga: α-Galactosidase
AcME: Acetylmannan esterase
BGL: β-Glucosidase
CAZy: Carbohydrate Active enZymes database
CAZyme: Carbohydrate-active enzyme
CBM: Carbohydrate binding module
CE: Carbohydrate esterase
DP: Degree of polymerisation
DS: Degree of synergy
EC: Enzyme commission number
EXP: Expansin
GalM: Galactomannan
GGM: Galactoglucomannan
GG: Guar gum
Glc: Glucose
GM: Glucomannan
GM1: β-D-Glc*p*-(1->4)-D-Man*p*
GM2: β-D-Glc*p*-(1->4)-β-D-Man*p*-(1->4)-D-Man*p*
GH: Glycoside hydrolase
HM: hetero-mannan
KGM: konjac glucomannan
hsHM: high galactose-substituted hetero-mannan
LBG: Locust bean gum
LPMO: Lytic polysaccharide monooxygenase
lsHM: low galactose-substituted hetero-mannan
M1: Mannose
M2: Mannobiose
M3: Mannotriose
M4: Mannotetraose
M5: Mannopentaose
M6: Mannohexaose
Man: Mannose
MAN: β-Mannanase
MBH: β-Mannobiohydrolase
Mnd: β-Mannosidase
MOS: Mannooligosaccharide(s)
RS: Reducing sugar(s)
Swol: Swollenin

## References

[CR1] Agger JW, Isaksen T, Várnai A (2014). Discovery of LPMO activity on hemicelluloses shows the importance of oxidative processes in plant cell wall degradation. Proc Natl Acad Sci USA.

[CR2] Andberg M, Penttilä M, Saloheimo M (2015). Swollenin from *Trichoderma reesei* exhibits hydrolytic activity against cellulosic substrates with features of both endoglucanases and cellobiohydrolases. Bioresour Technol.

[CR3] Anisha GS (2022) Microbial alpha-galactosidases: efficient biocatalysts for bioprocess technology. Bioresour Technol 344 10.1016/j.biortech.2021.126293 34752888

[CR4] Armstrong Z, Davies GJ (2020). Structure and function of Bs164 β-mannosidase from *Bacteroides salyersiae* the founding member of glycoside hydrolase family GH164. J Biol Chem.

[CR5] Bååth AJ, Martínez-Abad A, Berglund J et al (2018) Mannanase hydrolysis of spruce galactoglucomannan focusing on the influence of acetylation on enzymatic mannan degradation. Biotechnol Biofuels 11. 10.1186/s13068-018-1115-y 10.1186/s13068-018-1115-y PMC5907293 29713374

[CR6] Bågenholm V, Reddy SK, Bouraoui H (2016). Galactomannan catabolism conferred by a polysaccharide utilisation locus of *Bacteroides ovatus* : enzyme synergy and crystal structure of a β-mannanase. J Biol Chem jbc.

[CR7] Bågenholm V, Wiemann M, Reddy SK (2019). A surface-exposed GH26 -mannanase from *Bacteroides ovatus*: structure, role, and phylogenetic analysis of BoMan26B. J Biol Chem.

[CR8] Bai Y, Zhou X, Li N et al (2021) In vitro fermentation characteristics and fiber-degrading enzyme kinetics of cellulose, arabinoxylan, β-glucan and glucomannan by pig fecal microbiota. Microorganisms 9. 10.3390/microorganisms9051071 10.3390/microorganisms9051071 PMC8156825 34065679

[CR9] Berglund J, Azhar S, Lawoko M (2019). The structure of galactoglucomannan impacts the degradation under alkaline conditions. Cellulose.

[CR10] Berglund J, Mikkelsen D, Flanagan BM (2020). Wood hemicelluloses exert distinct biomechanical contributions to cellulose fibrillar networks. Nat Commun.

[CR12] Beukes N, Pletschke BI (2011). Effect of alkaline pre-treatment on enzyme synergy for efficient hemicellulose hydrolysis in sugarcane bagasse. Bioresour Technol.

[CR11] Beukes N, Chan H, Doi RH, Pletschke BI (2008). Synergistic associations between *Clostridium cellulovorans* enzymes XynA, ManA and EngE against sugarcane bagasse. Enzyme Microb Technol.

[CR13] Bhattacharya A, Wiemann M, Stålbrand H (2021) β-Mannanase BoMan26B from *Bacteroides ovatus* produces mannan-oligosaccharides with prebiotic potential from galactomannan and softwood β-mannans. 10.1016/j.lwt.2021.112215. LWT 151:

[CR14] Bi R, Berglund J, Vilaplana F (2016). The degree of acetylation affects the microbial degradability of mannans. Polym Degrad Stab.

[CR15] Biely P (2012). Microbial carbohydrate esterases deacetylating plant polysaccharides. Biotechnol Adv.

[CR16] Boraston AB, Bolam DN, Gilbert HJ, Davies GJ (2004). Carbohydrate-binding modules: fine-tuning polysaccharide recognition. Biochem J.

[CR17] Busch A, Danchin EGJ, Pauchet Y (2019) Functional diversification of horizontally acquired glycoside hydrolase family 45 (GH45) proteins in Phytophaga beetles. BMC Evol Biol 19. 10.1186/s12862-019-1429-9 10.1186/s12862-019-1429-9 PMC6509783 31077129

[CR18] Cairns JRK, Esen A (2010). β-Glucosidases. Cell Mol Life Sci.

[CR19] Cartmell A, Topakas E, Ducros VMa (2008). The *Cellvibrio japonicus* mannanase CjMan26C displays a unique exo-mode of action that is conferred by subtle changes to the distal region of the active site. J Biol Chem.

[CR20] Coconi Linares N, Dilokpimol A, Stålbrand H et al (2020) Recombinant production and characterization of six novel GH27 and GH36 α-galactosidases from *Penicillium subrubescens* and their synergism with a commercial mannanase during the hydrolysis of lignocellulosic biomass. Bioresour Technol 295. 10.1016/j.biortech.2019.122258 10.1016/j.biortech.2019.122258 31639625

[CR21] Couturier M, Roussel A, Rosengren A (2013). Structural and biochemical analyses of glycoside hydrolase families 5 and 26 β-(1,4)-mannanases from *Podospora anserina* reveal differences upon manno-oligosaccharide catalysis. J Biol Chem.

[CR22] Couturier M, Touvrey-Loiodice M, Terrapon N (2022). Functional exploration of the glycoside hydrolase family GH113. PLoS ONE.

[CR23] Daas PJH, Schols HA, De Jongh HHJ (2000). On the galactosyl distribution of commercial galactomannans. Carbohydr Res.

[CR24] Dea ICM, Clark AH, Mccleary B (1986). Effect of galactose-substitution-patterns on the inter-action properties of galactomannans. Carbohydr Res.

[CR25] Dhawan S, Kaur J (2007). Microbial mannanases: an overview of production and applications. Crit Rev Biotechnol.

[CR26] Dias FMV, Vincent F, Pell G (2004). Insights into the molecular determinants of substrate specificity in glycoside hydrolase family 5 revealed by the crystal structure and kinetics of *Cellvibrio mixtus* mannosidase 5A. J Biol Chem.

[CR27] Din N, Damude HG, Gilkes NR (1994). C1-Cx, revisited: intramolecular synergism in a cellulase. Proc Nati Acad Sci USA.

[CR28] Fanuel M, Garajova S, Ropartz D (2017). The *Podospora anserina* lytic polysaccharide monooxygenase PaLPMO9H catalyzes oxidative cleavage of diverse plant cell wall matrix glycans. Biotechnol Biofuels.

[CR29] Forsberg Z, Mackenzie AK, Sørlie M (2014). Structural and functional characterization of a conserved pair of bacterial cellulose-oxidizing lytic polysaccharide monooxygenases. Proc Natl Acad Sci U S A.

[CR30] von Freiesleben P, Moroz OV, Blagova E (2019). Crystal structure and substrate interactions of an unusual fungal β -mannanase from *Yunnania penicillata*. Sci Rep.

[CR31] Gourlay K, Hu J, Arantes V (2013). Swollenin aids in the amorphogenesis step during the enzymatic hydrolysis of pretreated biomass. Bioresour Technol.

[CR32] Gu X, Lu H, Chen W, Meng X (2021). Characterization of a novel thermophilic mannanase and synergistic hydrolysis of galactomannan combined with swollenin. Catalysts.

[CR33] Hägglund P (2002) Mannan-hydrolysis by hemicellulases Enzyme-polysaccharide interaction of a modular β-mannanase. PhD Thesis. Lund University

[CR34] Harjunpää V, Helin J, Koivula A (1999). A comparative study of two retaining enzymes of *Trichoderma reesei*: Transglycosylation of oligosaccharides catalysed by the cellobiohydrolase I, Cel7A, and the β-mannanase, Man5A. FEBS Lett.

[CR35] Herburger K, Franková L, Pičmanová M (2020). Hetero-trans-β-glucanase produces cellulose–xyloglucan covalent bonds in the cell walls of structural plant tissues and is stimulated by expansin. Mol Plant.

[CR36] Hsu Y, Arioka M (2020) In vitro and in vivo characterization of genes involved in mannan degradation in *Neurospora crassa*. Fungal Genet Biol 144 10.1016/j.fgb.2020.103441 32777385

[CR37] Jäger G, Wu Z, Garschhammer K (2010). Practical screening of purified cellobiohydrolases and endoglucanases with a-cellulose and specification of hydrodynamics. Biotechnol Biofuels.

[CR38] Jin Y, Petricevic M, John A (2016). A β-mannanase with a lysozyme-like fold and a novel molecular catalytic mechanism. ACS Cent Sci.

[CR39] Kaira GS, Kapoor M (2019). How substrate subsites in GH26 endo-mannanase contribute towards mannan binding. Biochem Biophys Res Commun.

[CR40] Kaira GS, Usharani D, Kapoor M (2019). Salt bridges are pivotal for the kinetic stability of GH26 endo-mannanase (ManB-1601). Int J Biol Macromol.

[CR41] Kalyani DC, Reichenbach T, Keskitalo MM (2021). Crystal structure of a homotrimeric verrucomicrobial exo-β-1,4-mannosidase active in the hindgut of the wood-feeding termite *Reticulitermes flavipes*. J Struct Biol X.

[CR42] Kawaguchi K, Ito S, Senoura T (2014). The mannobiose-forming exo-mannanase involved in a new mannan catabolic pathway in *Bacteroides fragilis*. Arch Microbiol.

[CR43] Kikuchi A, Okuyama M, Kato K (2017). A novel glycoside hydrolase family 97 enzyme: Bifunctional β-L-arabinopyranosidase/α-galactosidase from *Bacteroides thetaiotaomicron*. Biochimie.

[CR44] Kirsch R, Wielsch N, Vogel H (2012). Combining proteomics and transcriptome sequencing to identify active plant-cell-wall-degrading enzymes in a leaf beetle. BMC Genomics.

[CR45] Kirui A, Zhao W, Deligey F et al (2022) Carbohydrate-aromatic interface and molecular architecture of lignocellulose. Nat Commun 13. 10.1038/s41467-022-28165-3 10.1038/s41467-022-28165-3 PMC8795156 35087039

[CR46] Klyosov AA, Dotsenko GS, Hinz SWA, Sinitsyn AP (2012). Structural features of beta-(1,4)-d-galactomannans of plant origin as a probe for beta-(1,4)-mannanase polymeric substrate specificity. Carbohydr Res.

[CR47] Li F, Sun X, Yu W et al (2021) Enhanced konjac glucomannan hydrolysis by lytic polysaccharide monooxygenases and generating prebiotic oligosaccharides. Carbohydr Polym 253. 10.1016/j.carbpol.2020.117241 10.1016/j.carbpol.2020.117241 33278997

[CR48] Liu B, Krishnaswamyreddy S, Muraleedharan MN (2018). Side-by-side biochemical comparison of two lytic polysaccharide monooxygenases from the white-rot fungus *heterobasidion irregulare* on their activity against crystalline cellulose and glucomannan. PLoS ONE.

[CR49] Liu W, Ma C, Liu W (2021). Functional and structural investigation of a novel β-mannanase BaMan113A from *Bacillus* sp. N16-5. Int J Biol Macromol.

[CR50] Mafa MS, Pletschke BI, Malgas S (2021) Defining the frontiers of synergism between cellulolytic enzymes for improved hydrolysis of lignocellulosic feedstocks. Catalysts 11. 10.3390/catal11111343

[CR51] Mai-Gisondi G, Maaheimo H, Chong SL (2017). Functional comparison of versatile carbohydrate esterases from families CE1, CE6 and CE16 on acetyl-4-O-methylglucuronoxylan and acetyl-galactoglucomannan. Biochim Biophys Acta Gen Subj.

[CR55] Malgas S, van Dyk JS, Pletschke BI (2015a) A review of the enzymatic hydrolysis of mannans and synergistic interactions between β-mannanase, β-mannosidase and α-galactosidase. World J Microbiol Biotechnol 31. 10.1007/s11274-015-1878-2 10.1007/s11274-015-1878-2 26026279

[CR56] Malgas S, van Dyk SJ, Pletschke BI (2015b) β-Mannanase (Man26A) and α-galactosidase (Aga27A) synergism - A key factor for the hydrolysis of galactomannan substrates. Enzyme Microb Technol 70. 10.1016/j.enzmictec.2014.12.007 10.1016/j.enzmictec.2014.12.007 25659626

[CR52] Malgas S, Chandra R, van Dyk JS et al (2017) Formulation of an optimized synergistic enzyme cocktail, HoloMix, for effective degradation of various pre-treated hardwoods. Bioresour Technol 245. 10.1016/j.biortech.2017.08.186 10.1016/j.biortech.2017.08.186 28892706

[CR53] Malgas S, Mafa MS, Mkabayi L, Pletschke BI (2019) A mini review of xylanolytic enzymes with regards to their synergistic interactions during hetero-xylan degradation. World J Microbiol Biotechnol 35. 10.1007/s11274-019-2765-z 10.1007/s11274-019-2765-z 31728656

[CR54] Malgas S, Thoresen M, Moses V (2022). Analysis of the galactomannan binding ability of β-mannosidases, BtMan2A and CmMan5A, regarding their activity and synergism with a β-mannanase. Comput Struct Biotechnol J.

[CR57] Martínez-Abad A, Jiménez-Quero A, Wohlert J (2020). Influence of the molecular motifs of mannan and xylan populations on their recalcitrance and organization in spruce softwoods. Green Chem.

[CR59] Miao T, Basit A, Wen J (2021). High efficient degradation of glucan/glucomannan to cello-/mannan-oligosaccharide by endoglucanase via tetrasaccharide as intermediate. Food Chem.

[CR60] Michalak L, Leanti S, Rosa L (2020). A pair of esterases from a commensal gut bacterium remove acetylations from all positions on complex β-mannans. Proc Natl Acad Sci USA.

[CR61] Mikkelson A, Maaheimo H, Hakala TK (2013). Hydrolysis of konjac glucomannan by *Trichoderma reesei* mannanase and endoglucanases Cel7B and Cel5A for the production of glucomannooligosaccharides. Carbohydr Res.

[CR62] Moreira LRS, Filho EXF (2008). An overview of mannan structure and mannan-degrading enzyme systems. Appl Microbiol Biotechnol.

[CR63] Njokweni AP, Rose SH, van Zyl WH (2012). Fungal β-glucosidase expression in *Saccharomyces cerevisiae*. J Ind Microbiol Biotechnol.

[CR64] Olver B, van Dyk JS, Beukes N, Pletschke BI (2011). Synergy between EngE, XynA and ManA from *Clostridium cellulovorans* on corn stalk, grass and pineapple pulp substrates. 3 Biotech.

[CR65] Pawar PMA, Koutaniemi S, Tenkanen M, Mellerowicz EJ (2013). Acetylation of woody lignocellulose: significance and regulation. Front Plant Sci.

[CR66] Petrović DM, Várnai A, Dimarogona M (2019). Comparison of three seemingly similar lytic polysaccharide monooxygenases from *Neurospora crassa* suggests different roles in plant biomass degradation. J Biol Chem.

[CR67] Reddy SK, Bågenholm V, Pudlo NA (2016). A β-mannan utilization locus in *Bacteroides ovatus* involves a GH36 α-galactosidase active on galactomannans. FEBS Lett.

[CR68] Sachslehner A, Foidl G, Foidl N (2000). Hydrolysis of isolated coffee mannan and coffee extract by mannanases of Sclerotium rolfsii. J Biotechnol.

[CR69] Saito M, Nakaya M, Kondo T et al (2022) Gelation of konjac glucomannan by acetylmannan esterases from *aspergillus oryzae*. Enzyme Microb Technol 160. 10.1016/j.enzmictec.2022.110075 10.1016/j.enzmictec.2022.110075 35691189

[CR70] Shallom D, Shoham Y (2003). Microbial hemicellulases. Curr Opin Microbiol.

[CR71] Shi P, Yao G, Cao Y (2011). Cloning and characterization of a new β-mannosidase from *Streptomyces* sp. S27. Enzyme Microb Technol.

[CR72] Shimizu M, Kaneko Y, Ishihara S (2015). Novel β-1, 4-mannanase belonging to a new glycoside hydrolase family in *aspergillus nidulans*. J Biol Chem.

[CR73] Shoseyov O, Shani Z, Levy I (2006). Carbohydrate binding modules: biochemical properties and novel applications. Microbiol Mol Biol Rev.

[CR74] Srivastava PK, Kapoor M (2017). Production, properties, and applications of endo-β-mannanases. Biotechnol Adv.

[CR75] Sun XB, Gao DY, Cao JW et al (2023) BsLPMO10A from *Bacillus subtilis* boosts the depolymerization of diverse polysaccharides linked via β-1,4-glycosidic bonds. Int J Biol Macromol 230. 10.1016/j.ijbiomac.2023.123133 10.1016/j.ijbiomac.2023.123133 36621733

[CR77] Tailford LE, Money VA, Smith NL (2007). Mannose foraging by *Bacteroides thetaiotaomicron*: structure and specificity of the β-mannosidase, BtMan2A. J Biol Chem.

[CR76] Tailford LE, Ducros VMa, Flint JE (2009). Understanding how diverse β-mannanases recognize heterogeneous substrates. Biochemistry.

[CR78] Tarasov D, Leitch M, Fatehi P (2018). Lignin-carbohydrate complexes: Properties, applications, analyses, and methods of extraction: a review. Biotechnol Biofuels.

[CR79] Tenkanen M, Thornton J, Viikari L (1995). An acetylglucomannan esterase of *aspergillus oryzae*; purification, characterization and role in the hydrolysis of O-acetyl-galactoglucomannan. J Biotechnol.

[CR80] Tsukagoshi H, Nakamura A, Ishida T (2014). The GH26 β-mannanase RsMan26H from a symbiotic protist of the termite *Reticulitermes speratus* is an endo-processive mannobiohydrolase: heterologous expression and characterization. Biochem Biophys Res Commun.

[CR81] Tsukagoshi H, Nakamura A, Ishida T (2014). The GH26 β-mannanase RsMan26H from a symbiotic protist of the termite *Reticulitermes speratus* is an endo-processive mannobiohydrolase: heterologous expression and characterization. Biochem Biophys Res Commun.

[CR82] Uechi K, Watanabe M, Fujii T (2020). Identification and biochemical characterization of major β-mannanase in *Talaromyces cellulolyticus* mannanolytic system. Appl Biochem Biotechnol.

[CR58] McCleary B, Clark AH, Dea ICM, Rees DA (1985). The fine structures of carob and guar galactomannans. Carbohydr Res.

[CR83] van Dyk JS, Pletschke BI (2012). A review of lignocellulose bioconversion using enzymatic hydrolysis and synergistic cooperation between enzymes-factors affecting enzymes, conversion and synergy. Biotechnol Adv.

[CR84] van Zyl WH, Rose SH, Trollope K, Görgens JF (2010). Fungal β-mannanases: Mannan hydrolysis, heterologous production and biotechnological applications. Process Biochem.

[CR85] Várnai A, Huikko L, Pere J (2011). Synergistic action of xylanase and mannanase improves the total hydrolysis of softwood. Bioresour Technol.

[CR86] Venegas FA, Koutaniemi S, Langeveld SMJ (2022). Carbohydrate esterase family 16 contains fungal hemicellulose acetyl esterases (HAEs) with varying specificity. N Biotechnol.

[CR87] von Freiesleben P, Spodsberg N, Blicher TH (2016). An *aspergillus nidulans* GH26 endo-β-mannanase with a novel degradation pattern on highly substituted galactomannans. Enzyme Microb Technol.

[CR88] von Freiesleben P, Spodsberg N, Stenbæk A (2018). Boosting of enzymatic softwood saccharification by fungal GH5 and GH26 endomannanases. Biotechnol Biofuels.

[CR89] Xie J, He Z, Wang Z (2019). Efficient expression of a novel thermophilic fungal β-mannosidase from *Lichtheimia ramosa* with broad-range pH stability and its synergistic hydrolysis of locust bean gum. J Biosci Bioeng.

[CR90] Xu C, Xia T, Peng H (2023). BsEXLX of engineered *Trichoderma reesei* strain as dual-active expansin to boost cellulases secretion for synergistic enhancement of biomass enzymatic saccharification in corn and Miscanthus straws. Bioresour Technol.

[CR91] Yamabhai M, Sak-Ubol S, Srila W, Haltrich D (2016). Mannan biotechnology: from biofuels to health. Crit Rev Biotechnol.

[CR92] Yao Q, Sun TT, Liu WF, Chen GJ (2008). Gene cloning and heterologous expression of a novel endoglucanase, swollenin, from *Trichoderma pseudokoningii* S38. Biosci Biotechnol Biochem.

[CR93] Yennawar NH, Li L-C, Dudzinski DM (2006). Crystal structure and activities of EXPB1 (Zea m 1), a-expansin and group-1 pollen allergen from maize. Proc Natl Acad Sci USA.

[CR94] Yeoman CJ, Han Y, Dodd D, Laskin AI, Sariaslani S, Gadd GM (2010). Thermostable enzymes as Biocatalysts in the Biofuel Industry. Advances in Applied Microbiology.

[CR95] You X, Qin Z, Li YX (2018). Structural and biochemical insights into the substrate-binding mechanism of a novel glycoside hydrolase family 134 β-mannanase. Biochim Biophys Acta Gen Subj.

[CR96] You X, Qin Z, Yan Q (2018). Structural insights into the catalytic mechanism of a novel glycoside hydrolase family 113 -1,4-mannanase from *Amphibacillus xylanus*. J Biol Chem.

[CR97] Yu L, Lyczakowski JJ, Pereira CS (2018). The patterned structure of galactoglucomannan suggests it may bind to cellulose in seed mucilage. Plant Physiol.

[CR98] Zhang Y, Ju J, Peng H (2008). Biochemical and structural characterization of the intracellular mannanase AaManA of *Alicyclobacillus acidocaldarius* reveals a novel glycoside hydrolase family belonging to clan GH-A. J Biol Chem.

[CR99] Zhu X, Xin S, Ding H (2022). Functional characterization of a noncatalytic protein, Athe_0181, from *Caldicellulosiruptor bescii* in promoting Lignocellulose Hydrolysis. BioResources.

